# The Effect of Fuzheng Yiai Decoction on the Transdifferentiation of Lung Adenocarcinoma in EGFR-TKI-Resistant Mice

**DOI:** 10.1155/carj/8827810

**Published:** 2024-12-19

**Authors:** Jianfeng Sun, Danqing Luo, Hui Li, Di Zhang, Yesha Liu, Song Jin, Hong Guo, Chengshi He, Zhipeng Wu

**Affiliations:** ^1^Department of Rehabilitation, Hospital of Chengdu University of Traditional Chinese Medicine, Chengdu 610072, China; ^2^Sichuan Acupuncture and Moxibustion School, Chengdu 610097, China

**Keywords:** drug resistance, EGFR-TKIs, Fuzheng Yiai Decoction, lung adenosquamous carcinoma, transdifferentiation

## Abstract

This study aimed to investigate the effect of Fuzheng Yiai Decoction (FZYA) on epidermal growth factor receptor-tyrosine kinase inhibitor (EGFR-TKI) drug resistance in lung adenosquamous carcinoma (ASC). The expression of thyroid transcription factor 1 (TTF1) and p63 in tumor cells was observed by immunofluorescence staining. Meanwhile, 25 nude mice successfully inoculated with the human lung ASC cell line NCI-H596 were randomly divided into five groups, namely, the model, gefitinib, low-, medium-, and high-dose FZYA with gefitinib groups. After four weeks of daily intragastric administration of the various treatments, the tumor weight and volume inhibition rates were calculated. The positive expression rate and protein expression of TTF1 and p63 in mouse tumor tissues were evaluated by immunohistochemistry and western blot assays. The results showed that the adenocarcinoma part and the squamous cell carcinoma part of the lung tissue were not either one or the other, and each had unique biological behavior patterns. In terms of the tumor volume and weight, gefitinib treatment along with FZYA reduced the acquired resistance of EGFR-TKIs in lung ASC, and its inhibitory effect was superior to EGFR-TKI (gefitinib) treatment alone. Moreover, it was discovered that FZYA inhibited the pedigree transformation among cancer subtypes due to EGFR-TKI treatment. In conclusion, the application of FZYA inhibited the pedigree transformation among lung cancer subtypes, thus increasing the tumor inhibitory effect and decreasing the EGFR-TKI drug resistance of tumor cells.

## 1. Introduction

Lung cancer is one of the most common malignant tumor types worldwide [1]. According to the histological categorization of lung and pleural tumors provided by the World Health Organization, lung adenosquamous carcinoma (ASC) is a rare and distinct pathological subtype characterized by a high degree of malignancy. This subtype exhibits histological characteristics that are indicative of both lung adenocarcinoma (ADC) and lung squamous carcinoma (SCC). Whether there exists lineage correlation or dynamic transition between lung ADC and SCC in ASC is a fundamental yet unsolved question, which is not only important for a better understanding of lung cancer biology but also potentially helpful for lung cancer therapies in the clinic. However, the paucity of matched clinical specimens at different stages from the same patients makes it difficult to examine the dynamic transformation of lung cancer. Therefore, studies using genetically engineered mouse models of human lung cancer are urgently needed.

Many patients with ASC are already at an advanced stage when diagnosed and have lost the opportunity for surgery [2]. Therefore, pharmacotherapy plays an important role in the comprehensive treatment of lung cancer. However, the efficacy rates of platinum-based combination chemotherapy for NSCLC are only 30%–40%, and the median survival time is 8–11 months [3]. Besides, its adverse effects are serious and intolerable.

In previous studies, patients diagnosed with EGFR mutation-positive SCC through small biopsy specimens were often reclassified as ASC or poorly differentiated ADC based on the surgical specimens collected postoperatively [4]. Researchers also have applied next-generation sequencing, fluorescence in situ hybridization, and laser capture microdissection techniques to further clarify the molecular characteristics of different cellular components in ASC [5]. Furthermore, an analysis of tissue samples from 16 Caucasian ASC patients revealed a significantly higher incidence of EGFR mutations in ASC patients compared to those with pure ADC or SCC [6]. Clinical trial data are consistent with these findings. Among 140 lung cancer patients, 46 had EGFR mutations, yielding a mutation rate of 33%. The mutation rates for adenocarcinoma, ASC, SCC, and large-cell carcinoma were 50.6%, 87.5%, 2.5%, and 5.3%, respectively [7]. Another study involving 52 NSCLC patients identified somatic mutations in the EGFR tyrosine kinase domain in 10 cases. Moreover, the mutation rates for ADC (26.1%) and ASC (40%) were significantly greater than that for SCC (0%) [8]. Additionally, research indicates that EGFR mutations are predominantly associated with ASC, with a mutation rate of 70.2% among ASC cases. These findings align with a previous large-scale clinical analysis [9], further supporting the conclusion that EGFR mutations are highly prevalent in ASC. This evidence provides valuable insights into the molecular mechanisms of ASC and informs therapeutic strategies for patients with EGFR mutations.

Gefitinib, a first-generation epidermal growth factor receptor-tyrosine kinase inhibitor (EGFR-TKI), is commonly used as a first-line treatment for EGFR-mutant lung cancers due to its effectiveness in the initial treatment phase [10]. Currently, gefitinib is the most widely used medication among patients as it is the most frequently recommended by healthcare professionals, accounting for the highest share of medical expenses. Despite its efficacy, resistance to gefitinib typically develops within 9–13 months of treatment, significantly limiting its long-term effectiveness [11]. Extensive research has been conducted on the mechanisms underlying gefitinib resistance, but no breakthrough progress has been achieved until now. These challenges highlight the need for further research into the molecular mechanisms driving EGFR-related resistance in ASC to optimize therapeutic strategies and to improve patient outcomes. Therefore, this study aimed to conduct specialized and in-depth research on the mechanisms of gefitinib resistance by exploring the use of traditional Chinese medicine (TCM) to inhibit gefitinib resistance in patients, which holds significant clinical value.

TCM has increasingly become a popular treatment strategy for cancer patients. TCM can relieve the clinical symptoms and treatment-related complications of several cancer types, improve the quality of life of patients, and reduce the side effects of conventional treatment. Fuzheng Yiai Decoction (FZYA) is a TCM formulation composed of seven herbs that is widely recognized for its role in enhancing the efficacy of cancer therapies. It has been used for many years at the Hospital of Chengdu University of TCM for the treatment of NSCLC. FZYA has been traditionally used to strengthen the immune system and to improve the overall health of patients [12]. Recent studies have demonstrated that FZYA can effectively enhance the therapeutic effects of EGFR-TKIs in lung cancer treatment. For instance, clinical observations [13] have revealed that combining FZYA with gefitinib not only improved the tumor control rate and reduced drug resistance compared to gefitinib alone but also alleviated treatment-related adverse effects such as diarrhea, rash, and nausea. These findings suggest that FZYA may inhibit tumor growth and modulate the tumor microenvironment through its anti-inflammatory and immunoregulatory properties. In our previous study [14], among 41 patients with advanced lung ASC treated with gefitinib with or without FZYA, the combination group demonstrated an efficacy rate of 28.57% and a tumor control rate of 61.90%, compared to 25.00% and 55.00%, respectively, in the gefitinib-only group. Furthermore, the improvement in life quality (Karnofsky score) was higher in the combination group (85.7%) than in the gefitinib group (70%), with a lower incidence of adverse effects. These findings underscore the potential of FZYA in lung cancer treatment.

This study focused on gefitinib due to its extensive clinical use and well-documented resistance mechanisms, which provide a robust foundation for investigating the potential of FZYA to enhance treatment efficacy. While other EGFR-TKIs may exhibit distinct resistance profiles, gefitinib serves as a representative model to explore how FZYA impacts EGFR-TKI resistance and tumor subtype transdifferentiation. This study holds significant clinical value and offers the potential to provide new therapeutic strategies with an enhanced treatment efficacy and improved patient outcomes.

## 2. Materials and Methods

### 2.1. Preparation of FZYA and EGFR-TKI (Gefitinib)

FZYA is composed of raw *Astragalus membranaceus* (60 g), *Panax ginseng* (15 g), *Fritillaria thunbergii* (30 g), *Cornus officinalis* (20 g), cooked *Rehmannia glutinosa* (20 g), fried *Atractylodes macrocephala* (20 g), and *Psoralea corylifolia* (20 g). All herbs were purchased from the Hospital of Chengdu University of Traditional Chinese Medicine.

The herbs were immersed in 1000 mL of distilled water, soaked for 30 min, boiled at high heat, and then simmered at low heat for an additional 60 min. The resulting decoction was collected, concentrated, and cooled. The density of the concentrated liquid was measured using Pome's hydrometer, and the liquid was adjusted appropriately for the low-, medium-, and high-dose groups.

Based on the conversion coefficient between human and nude mouse drug doses (9.1) [15], the daily dosage for an adult human weighing 60 kg is 18 g [16], which is equivalent to 0.3 g/kg. This converts to a daily dosage of 2.73 g/kg for nude mice. Given that the average weight of a nude mouse is approximately 20 g, the daily dose per mouse was calculated to be 0.0546 g. Since each mouse received a daily dose of 0.2 mL, with 0.273 g of Chinese medicine dissolved in each mL of water, the resulting concentration of 1.273 g/mL (1 g water + 0.273 g of Chinese medicine per mL) was designated as the medium-dose group. The low-dose group concentration was set at half of the medium dose (1.15 g/mL), while the high-dose group concentration was twice the medium dose (1.6 g/mL).

The standard daily dose of gefitinib for humans is 250 mg. Using a human-to-mouse conversion factor of 9.1 [15], the equivalent daily dose for each nude mouse was calculated to be 0.76 mg. In this study, intragastric administration for each mouse was set at 0.2 mL, with this volume containing 0.76 mg of gefitinib.

### 2.2. Animal Experiments

A total of 25 male BALB/c nude (nu/nu) mice (4 weeks old, 20 g) were purchased from Chengdu Dossy Experimental Animals Co., Ltd. (Certificate No. SCXK (Chuan) 2020-030, Sichuan, China). All mice were reared by the Experimental Animal Center of West China Medical Center, Sichuan University (Qualification No. SYXK (Chuan) 2018-119), and were specific-pathogen-free grade. All mice were fed under normal conditions and housed at a temperature of 20°C–26°C and a relative humidity of 50%–60%, with ventilation. The animal experimental protocols were approved by the Animal Care and Use Committee of the Hospital of Chengdu University of Traditional Chinese Medicine (Certificate No. 2020DL-003).

The human lung ASC cell line NCI-H596 (ATCC No. HTB-178, Lot No. 63821792) used in this study was maintained by the Biotherapy National Key Laboratory of West China Medical Center, Sichuan University. After sufficient subculturing, NCI-H596 cells in the logarithmic phase of growth were digested by 0.25% trypsin and made into a single cell suspension. A 0.2-mL aliquot of the single cell suspension (1 × 10^7^ cells) was injected subcutaneously into the right inguinal region of each mouse. At six weeks after the injection, 25 nude mice were randomly divided into five groups (five mice per group), namely, the model (gavage with 0.4 mL of saline), gefitinib (gavage with 0.2 mL of gefitinib and 0.2 mL of saline), low-dose FZYA combined with gefitinib (gavage with 0.2 mL of gefitinib and 0.2 mL of 1.15 g/mL FZYA), medium-dose FZYA combined with gefitinib (gavage with 0.2 mL of gefitinib and 0.2 mL of 1.3 g/mL FZYA), and high-dose FZYA combined with gefitinib (gavage with 0.2 mL of gefitinib and 0.2 mL of 1.6 g/mL FZYA). All mice were treated for four weeks.

### 2.3. Immunofluorescence Staining

The human lung ASC cells were subcultured and prepared onto slides. After being washed three times with phosphate-buffered saline (PBS), the cells on the prepared slides were fixed with 4% paraformaldehyde (200 μL/hole) for 15 min at room temperature, washed three times with PBS (5 min each), then lysed with 0.1% Triton X-100 for 10 min, and washed three times with PBS (5 min each). The slides were then blocked with 5% bovine serum albumin (BSA) for 1 h at room temperature and incubated overnight at 4°C with primary antibody (TTF1 :1/500, P63: 1/300, Abcam, USA). After washing the slides three times with PBS (5 min each), the slides were incubated with 5% BSA in the dark (1 h) and then with fluorescent secondary antibody solution (1/500) at room temperature for 1 h. After washing the slides three times with PBS (5 min each), the slides were mounted with 0.1 μg/mL 4′,6-diamidino-2-phenylindole and then observed under a fluorescence microscope.

### 2.4. Measurement of Tumor Volume and Weight

The nude mice were sacrificed after the experiment was completed, the tumor tissues were stripped on a clean bench, the tumor length was measured with a Vernier caliper, and the tumor weight was determined with an electronic balance. The volume of the tumors was calculated using the following formula: volume of tumor = (longest diameter × shortest diameter^2^)/2. The average tumor inhibition rate was calculated using the following formulas: tumor volume inhibition rate (%) = (the average tumor volume of the model group − the average tumor volume of the respective experimental group)/the average tumor volume of the model group × 100%; tumor weight inhibition rate (%) = (the average tumor weight of the model group − the average tumor weight of the respective experimental group)/the average tumor weight of the model group × 100%.

### 2.5. Immunohistochemical Staining

The tumor tissue was washed with saline, blocked with 4% polyoxymethylene, and treated with dimethylbenzene, alcohol, and ceresin wax. Next, the tumor tissue was made into sections (5 μm). The sections were washed and then soaked in 3% hydrogen peroxide for 10 min to eliminate endogenous enzymes. The sections were cooled with flowing water after they were steamed for 3 min (two times) in retrieval solution, and then they were washed with PBS for 3 min (three times) to restore the tissue antigens. The sections were blocked with BSA at 37°C for 30 min after drying and were then incubated with primary antibody (TTF1: 1/250, P63: 1/1200, Abcam, USA) at 4°C overnight. After washing with PBS for 10 min (three times), the sections were incubated with horseradish peroxidase (HRP)–conjugated (Abcam, USA) secondary antibody (1/500) at 4°C for 2 h. After washing the sections with PBS for 10 min (three times) and preparing the diaminobenzidine (DAB) chromogenic agent according to the manufacturer's instructions, DAB, hydrogen peroxide, and PBS were added to the sections in order, and then the sections were washed for 10 min after developing for 10 min. The sections were washed with flowing water for 2 min after being dyed with hematoxylin (Solarbio, China), then treated with acid alcohol, washed with flowing water, dried, and sealed. Five fields were selected on each section and observed under a microscope (Nikon, Japan). The images were processed with ImageJ software, which recorded the amount of positive tumor cells in the fields, then the ratio of positive tumor cells to all tissue cells was assessed, and the total amount of positive tumor cells was calculated based on the result of the tumor weight.

### 2.6. Western Blot Analysis

The tumor tissues were stripped on a clean bench, washed with PBS, and then transferred into a homogenizer to smash and pyrolyze the tissues with the proper amount of radioimmunoprecipitation assay (RIPA) lysis buffer (Pierce, USA). After complete lysing and centrifugation (4°C, 12,000 rpm for 30 min), the supernatant was collected. The protein concentration was calculated by a bicinchoninic acid protein assay kit (Beyotime, China). The protein samples were mixed with RIPA lysis buffer and then kept at −20°C after denaturation for 5 min in a 100°C water bath (Yiheng, Shanghai, China). Electrophoresis was carried out in the concentration gel at a constant voltage of 50 V and in the separation gel at a constant current of 20 mA until the bromophenol blue indicator reached the lower margin of the plastic board. The protein gel tape needed was cut off, then a suitable size of polyvinylidene fluoride (PVDF) membrane (Millipore, German) was tailored and soaked with carbinol. The protein samples were transferred to the membrane at a constant voltage of 50 V. Thereafter, the PVDF membrane was washed with tris-buffered saline containing 0.1% Tween (TBST) for 5 min and then blocked with BSA for 1 h at room temperature. The PVDF membrane was incubated with primary antibody (TTF1: 1/2000, P63: 1/2000, Abcam, USA) at 4°C overnight, followed by washing with TBST for 5 min (three times). The membrane was then incubated with secondary antibody (1/500), followed by washing with TBST for 5 min (three times). The membrane was coated with enhanced chemiluminescence reagents (1:1) for 3 min. The results were detected with an X-ray film developer (FUJIFILM, Japan). The X-ray film was scanned with an Epson perfection V39 scanner (Seiko Epson Corporation, Japan). The image was processed with ImageJ software.

### 2.7. Statistical Analysis

The statistical analysis of data was carried out using SPSS 22.0 statistical software. The measurement data were expressed as mean ± standard deviation, and skewed data were expressed as the median and interquartile range. Multiple mean comparisons among groups were performed with variance analysis. Pairwise comparisons among groups were performed with the independent-samples *t*-test. Enumeration data were expressed as a percentage. Comparisons among enumeration data were performed with the chi-squared test. *p* < 0.05 was considered statistically significant.

## 3. Results

### 3.1. Expression of TTF1 and p63 in Lung ASC Cells

**Figure 1:**
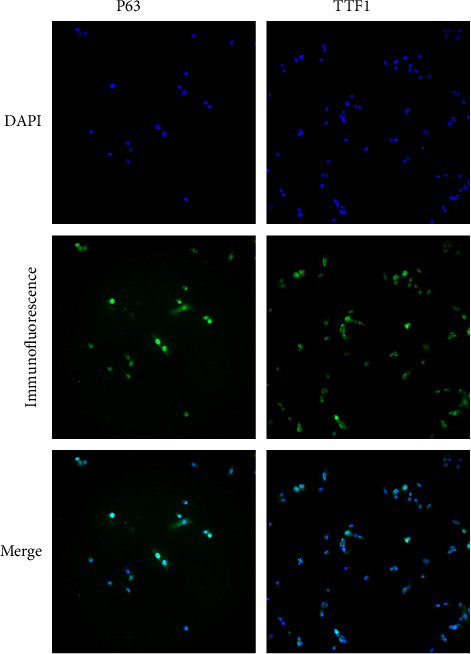
The expression of TTF1 and p63 in NCI-H596 cells by immunofluorescence staining.

The cells on slides after immunofluorescence staining were observed, and the locations of cellular TTF1 and p63 expression were recorded with a fluorescence microscope. The location of cellular TTF1 and p63 expression was observed mostly in the cell nucleus; it is worth noting that TTF1 is a specific antibody for lung ADC, while p63 is a specific antibody for lung SCC; NCI-H596 cells are special lung ASC cells that express TTF1 and p63 in the cell nucleus simultaneously (Figure 1).

### 3.2. Inhibitory Effect of FZYA Along With Gefitinib on Mouse Tumor Volume and Weight

Compared with the model group, the tumor volume and weight were significantly reduced in the mice treated with FZYA and/or gefitinib (*p* < 0.05). Treatment with FZYA along with gefitinib reduced the mouse tumor volume more obviously than that with gefitinib alone (*p* < 0.001). Furthermore, treatment with high- or medium-dose FZYA along with gefitinib reduced the mouse tumor volume more than low-dose FZYA along with gefitinib (*p* < 0.001). However, there was no significant difference between the medium- and high-dose FZYA groups (*p* > 0.05).

**Figure 2:**
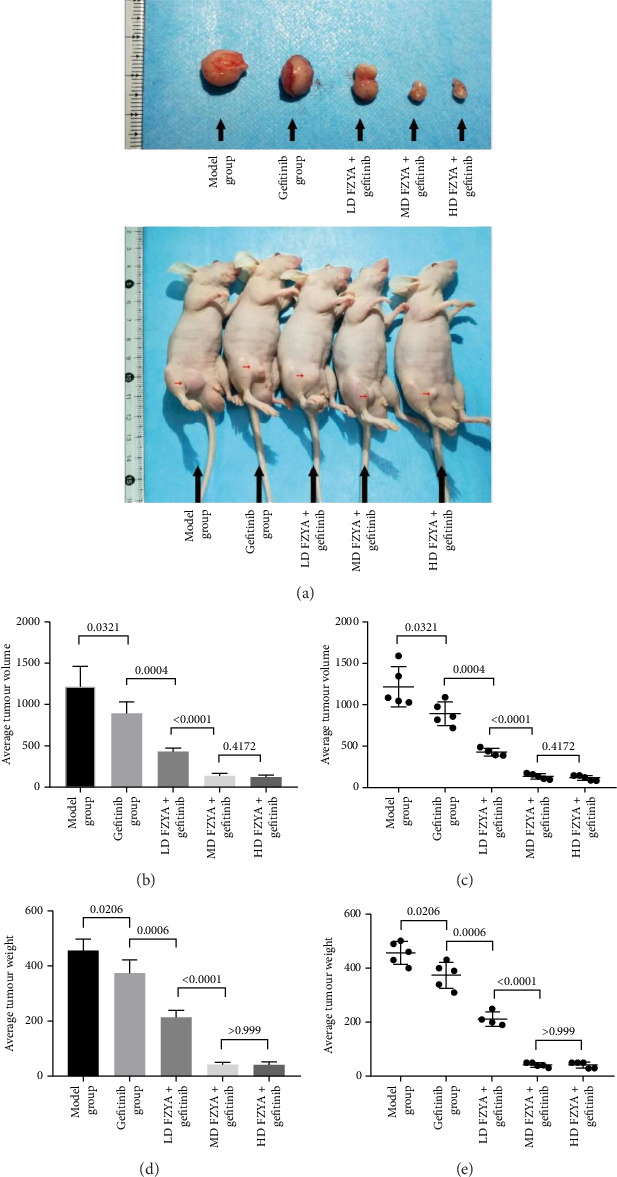
The tumor volume and tumor weight of each group. (a) The tumor status of each group. (b, c) The tumor volume of each group. (d, e) The tumor weight of each group.

Treatment with high-dose FZYA along with gefitinib exhibited the highest tumor volume (90.2%) and weight (90.8%) inhibition rates, followed by the medium- (88.8% and 90.8%, respectively) and low-dose FZYA (64.9% and 53.4%, respectively) groups. The gefitinib group showed the lowest tumor volume (20.8%) and weight (18.0%) inhibition rates, among the four treatment groups (Figure 2 and Tables 1 and 2).

### 3.3. Effects of FZYA Combined With Gefitinib on TTF1 and p63 Expression

**Figure 3:**
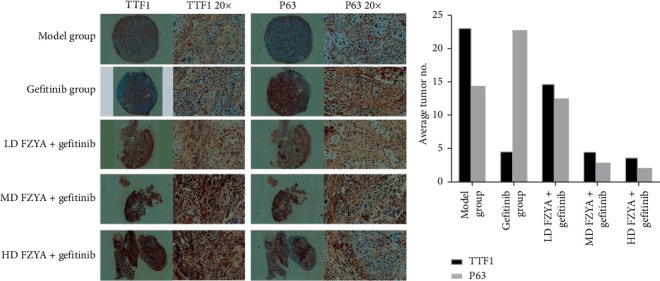
The expression of TTF1 and p63 in tumor tissues by immunohistochemical staining.

The expression of TTF1 and p63 in the tumors of mice treated with gefitinib and/or FZYA as assessed by immunohistochemical staining is shown in Figure 3. According to the results, treatment with gefitinib and/or FZYA significantly decreased the total numbers of TTF1-positive and p63-positive cells, among which the medium- and high-dose FZYA groups exhibited the strongest inhibitory effect (*p* < 0.05), followed by low-dose FZYA (*p* < 0.05). In contrast, the proportion of TTF1-positive and p63-positive cells in the total tumor cells in the treatment groups presented a decreasing trend compared with the model group. The medium- and high-dose FZYA groups showed the lowest proportion of positive cells, followed by the low-dose FZYA group (*p* < 0.05). Specifically, the high-dose FZYA group showed the highest expression ratio of TTF1 to p63 (1.71), followed by the model group (1.59), the medium-dose FZYA group (1.55), and the low-dose FZYA group (1.16). The gefitinib group showed the lowest expression ratio of TTF1 to p63 (0.20).

### 3.4. The Protein Expression of TTF1 and p63 in Mouse Tumor Tissues

**Figure 4:**
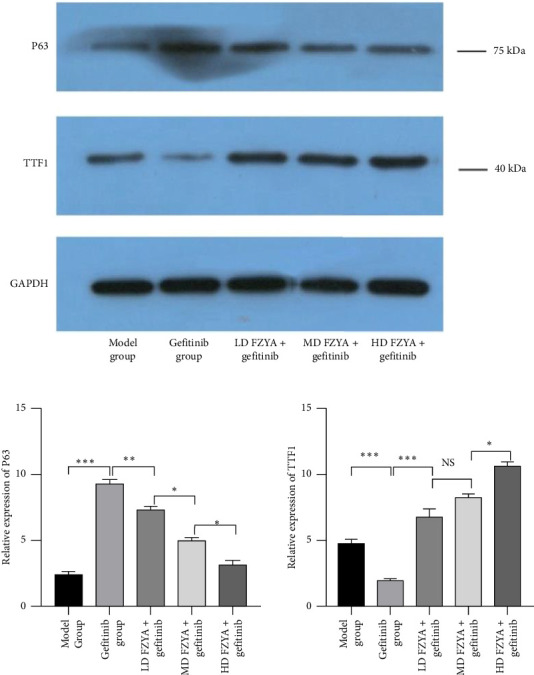
The protein expression of p63 and TTF1 in mouse tumor tissues by western blot. ⁣^∗^*p* < 0.05, ⁣^∗∗^*p* < 0.01, ⁣^∗∗∗^*p* < 0.001, versus the adjacent group.

According to the western blot images shown in Figure 4, treatment with gefitinib with or without FZYA obviously decreased the p63 protein expression compared to the model group (*p* < 0.05), among which the high-dose FZYA group displayed the strongest inhibitory effect on p63 expression. No difference between the low-dose and medium-dose FZYA groups was observed (*p* < 0.05). Besides, the TTF1 protein expression showed the opposite trend as the p63 expression. The high-dose FZYA group had the highest TTF1 protein expression (*p* < 0.05).

## 4. Discussion

Public interest and demand for complementary and alternative medicine services have increased over the last decade. TCM plays an important role in reducing disability, protecting cancer patients from complications, and reducing side effects of conventional treatments. FZYA has been used in the treatment of NSCLC for several years in the Hospital of Chengdu University of Traditional Chinese Medicine. However, the mechanism by which FZYA improves the treatment efficiency for advanced lung cancer remains unclear. At present, there are no reports on the effect of TCM on reducing drug resistance by regulating the transdifferentiation of tumor cells. Herein, this study suggests a novel mechanism of resistance to targeted drugs at the cellular level, which is different from the previously established molecular signaling pathway.

Phenotypic plasticity and functional heterogeneity are important features of various tumors [17–19]. Analyzing the underlying phenotypic plasticity and dynamic lineage correlations between tumor subtypes helps to explore tumorigenesis and to develop effective therapeutic strategies for cancers. Even though the histopathological heterogeneity of lung cancer has been established, the phenotypic plasticity among different subtypes of lung cancer is still not fully understood. A previous study [20] has identified a genotypic and histological transition from EGFR-mutated NSCLC to small-cell lung cancer (SCLC) after targeted drug therapy. NSCLC with the EGFR mutation transfers to SCLC according to its genotype and histology after targeted treatment. Mixed human lung tumors with the same gene mutation, ADC, and SCC are often observed in the same lesion. It is generally believed that lung ADC originates from the pedigree of type II pneumocytes, developing stepwise from atypical adenomatous hyperplasia [21, 22], while lung SCC may originate from basal cells [23]. The phenotypic plasticity of lung ADC has been reported in a previous study, revealing that lung ADC with a gene defect can gradually transdifferentiate into lung SCC [24]. Besides, an intense transdifferentiation process occurs in a phased and systematic manner. It is different from traditional cancer stem cells in which only a few cell populations are endowed with phenotypic plasticity [25]. Through lineage tracing and pathologic analysis of gene-deficient mice with lung cancer, it has been demonstrated that lung ADC of the mice could gradually differentiate into SCC through ASC cells as an intermediate. Hypoxia in ADC leads to the decreased expression of lysyl oxidase (Lox), induces extracellular matrix (ECM) remodeling, and turns on squamous gene expression, thereby upregulating p63 expression. Furthermore, previous studies have shown that from a pathological perspective, selectively inhibiting Lox can effectively suppress the generation of gene-deficient ADC and promote the transdifferentiation of ADC into SCC. Therefore, ADC and SCC are not equally sensitive to hypoxic ECM changes. The sensitivities of ADC and SCC to ECM changes caused by hypoxia are different. The specific regulatory pathway is the early stage of the gene-deficient lung ADC phenotype. The gene defect promotes the growth and development of ADC through the overexpression of Lox [26]. With the growth of the tumor, aggravation of the hypoxia level decreases the Lox expression and causes the remodeling of ECM, which leads to a sharp change in the intracellular environment. Ultimately, the ADC can be strongly and massively transformed into SCC. In addition, ADC metastasis could not be observed until 10 weeks after treatment, while ADC-to-SCC transdifferentiation could be observed only 8 weeks after treatment [24]. Therefore, it is excluded that transdifferentiation of the tumor phenotype is due to factors of tumor metastasis to other sites and that the two may be independent events during tumor progression. Recent studies also have indicated that multiple lineage transcription factors form antagonistic regulatory networks during the occurrence and development of lung ASC. If the regulation is out of balance, tumor cells will undergo a state of high immune cell infiltration and eventually achieve ASC transdifferentiation [27]. This differs from the previously established molecular mechanisms involved in the development of resistance and may be a unique and novel mechanism for circumventing drug therapy at the cellular level.

TTF1 and p63 are specific antibodies for lung ADC and SCC, respectively. In this study, lung ASC expressed both TTF1 and p63 in the nucleus. Evidence has revealed that lung ASC is not a simple mixture of ADC and SCC, but it has a more complex and unique molecular phenotype. It is also believed that lung ASC has a unique biological behavior. Its ADC and SCC parts originate from a common precursor and differentiate into different histomorphologies during disease progression. Therefore, the expression of TTF1 and p63 in NCI-H596 cells is not one or the other, which provides evidence for the transdifferentiation between lung cancer cells due to the drug treatment. Besides, the changes of tumor volume and weight were consistent in this study. Gefitinib combined with FZYA was more effective than gefitinib alone in inhibiting tumor growth. In addition, an increased FZYA concentration gradually increased the inhibitory effect on the tumor. However, when a certain threshold of the FZYA concentration was reached, its inhibitory effect on tumors no longer increased. Although the combined use of gefitinib and FZYA improved the tumor inhibitory effect, the absolute number of tumor cells decreased. Moreover, in the remaining tumor tissue, the density of tumor cells increased, which could also be considered as the process of tumor treatment, and the tumor cells were concentrated.

Furthermore, the western blot results were similar to those of the immunohistochemical staining. Treatment with gefitinib alone significantly reduced the proportion of lung ADCs, while it increased the proportion of lung SCCs. In contrast, treatment with gefitinib along with FZYA significantly increased the proportion of lung ADCs, while it decreased the proportion of lung SCCs. This observation was closely related to regulation of the potential lineage switching between these lung cancer cell subtypes. Transdifferentiation of cells in response to environmental factors has been shown to be beneficial to the environment in which the cells are located [28, 29].

Treatment with gefitinib altered the tumor microenvironment. The lineage conversion between lung cancer cell subtypes (ADC to SCC) occurred in order to adapt to these environmental changes. The heterogeneity of cancer cells was reflected at this time. Therefore, different subtypes of tumor cells may lead to differences in drug sensitivity.

FZYA inhibited the lineage switching between lung cancer cell subtypes, thereby improving the effect of inhibiting tumor growth and reducing the drug resistance of tumor cells. However, at present, we do not know which genes and signaling pathways are regulated by FZYA to achieve lineage conversion between lung cancer cell subtypes. As the next step, we will use genomics and proteomics technologies to explore the relationship between abnormal gene expression and the protein molecular signal network involved in this process as well as screen and establish relatively major protein molecular signaling pathways. This research will further elucidate the mechanism of the effect of FZYA prescription on EGFR-TKI resistance in lung ASC.

## 5. Conclusions

In conclusion, treatment with gefitinib along with FZYA inhibited the lineage switching between lung cancer cell subtypes, thereby improving the tumor inhibitory effect and reducing the drug resistance of tumor cells. This study unveils a novel molecular mechanism by which FZYA inhibits the growth of lung cancer cells. Nevertheless, more in-depth experiments, such as proteomics and pharmacological analysis, are required to further identify the active components and reveal the biological functions of FZYA.

## Figures and Tables

**Table 1:** The tumor volume and tumor volume inhibition rate status of each group.

Group	Number	Tumor volume (mm^3^)	Inhibition rate (%)
Model	5	1219.97 ± 243.16	—
Gefitinib	5	893.57 ± 142.54	26.8
FZYAL + gefitinib	4	428.79 ± 45.79	64.9
FZYAM + gefitinib	5	136.95 ± 33.37	88.8
FZYAH + gefitinib	5	119.92 ± 29.47	90.2

*Note:* Values are expressed as mean ± standard deviation. FZYAL, FZYAM, and FZYAH represent low-, medium-, and high-dose FZYA, respectively.

**Table 2:** The tumor weight and tumor weight inhibition rate status of each group.

Group	Number	Tumor weight (mg)	Inhibition rate (%)
Model	5	456.00 ± 41.59	—
Gefitinib	5	374.00 ± 48.27	18.0
FZYAL + gefitinib	4	212.50 ± 26.30	53.4
FZYAM + gefitinib	5	42.00 ± 8.37	90.8
FZYAH + gefitinib	5	42.00 ± 10.95	90.8

*Note:* Values are expressed as mean ± standard deviation. FZYAL, FZYAM, and FZYAH represent low-, medium-, and high-dose FZYA, respectively.

## Data Availability

The datasets generated and analyzed during the current study are available from the corresponding authors on reasonable request.

## Nomenclature

ADC: Adenocarcinoma
ASC: Adenosquamous carcinoma
BSA: Bovine serum albumin
DAB: Diaminobenzidine
ECM: Extracellular matrix
EGFR-TKI: Epidermal growth factor receptor-tyrosine kinase inhibitor
FZYA: Fuzheng Yiai Decoction
HRP: Horseradish peroxidase
Lox: Lysyl oxidase
NSCLC: Non-small-cell lung carcinoma
PBS: Phosphate-buffered saline
PVDF: Polyvinylidene fluoride
RIPA: Radioimmunoprecipitation assay
SCC: Squamous cell carcinoma
SCLC: Small-cell lung cancer
TBST: Tris-buffered saline containing 0.1% Tween
TTF1: Thyroid transcription factor 1
FZYAL: Low-dose FZYA
FZYAM: Medium-dose FZYA
FZYAH: High-dose FZYA
